# Intraperitoneal hypertension, a novel risk factor for sepsis-associated encephalopathy in sepsis mice

**DOI:** 10.1038/s41598-018-26500-7

**Published:** 2018-05-25

**Authors:** Yu-jing He, Hao Xu, Yao-jie Fu, Ji-yan Lin, Min-wei Zhang

**Affiliations:** 1 Intensive Care Unit, First Affiliated Hospital of Xiamen University, Xiamen, Fujian province China; 2 Emergency Department, First Affiliated Hospital of Xiamen University, Xiamen, Fujian province China

## Abstract

Sepsis associated encephalopathy (SAE), appears often indicates the deterioration of the sepsis disease and which have high risk of death. Although several mechanism and hypotheses have been proposed and studied, there is no breakthrough in the treatment of SAE. We performed a systematic research to evaluate the effect of intraperitoneal pressure on SAE. A mice model of sepsis was established by intraperitoneal injection of endotoxin. A total of 48 female BALB/c mouse (30 days old) were randomly divided into a control group (n = 12) and an injection of endotoxin referred to bacterial lipopolysaccharide (LPS) group (n = 12). Intraperitoneal hypertension (IAH) referred to IAH group (n = 12), and LPS + IAH group (n = 12). Following sepsis induction, diagnosis, the brains were analyzed for both function and ultrastructural morphology.We determined that IAH exacerbated sepsis induces sepsis-associated encephalopathy when examining low score of neurological function and more delta wave in EEG, increased neuronal edema in LPS + IAH group, as well as an escalation of Bax and Cleaved-caspase-3, Cleaved-parp, and reduction of Bcl-2 and Mfsd2a in LPS + IAH group. Therefore, IAH can exacerbate and increase incident rate of sepsis-related encephalopathy in sepsis mice by promoting neuronal apoptosis and destruction of the blood-brain barrier.

## Introduction

Sepsis is a serious threat to human health, epidemiological survey showed that the incidence of sepsis in the United States is 3‰, more than half need to be treated in ICU^1^. Each year about 750,000 cases are diagnosed as severe sepsis, of which 215,000 deaths in sepsis-related organ dysfunction^2,3^. In sepsis-induced systemic damage, the central nervous system is undoubtedly one of the most vulnerable organs, therefore one of the more severe clinical manifestations of sepsis is sepsis associated encephalopathy (SAE)^4,5^.

Although the pathogenesis of SAE has not yet fully understood, several mechanism has been proposed including reduced autonomic cerebrovascular function, microcirculation, endothelial cell activation, amino acid imbalance, mitochondrial dysfunction, glial cell function damage and neuronal apoptosis^6–8^. However, when all these mechanisms are linked and analyzed, we will find that all these pathophysiological processes cannot be separated from blood-brain barrier (BBB) destruction. BBB are regulated by a series of transporters, by tightly controlling the molecule and ion permeability, providing nutrients and oxygen instantly according to neuron needs, and expelling potential toxins, protecting the brain against pathogens and toxins intrusion, so that the steady-state neurons can be maintained to play a normal function^9^. Therefore, any cause of central nervous system dysfunction factors must first start from the destruction of BBB, sepsis-induced brain damage is no exception.

Studies have confirmed that there is indeed BBB injury in sepsis. In sepsis, BBB is damaged by various factors such as endotoxin, inflammatory factor, reactive oxygen species, complement, endothelial cell activation, glial cell activation and a large amount of dysregulated cytokines^10^. Cerebral edema causing by neurotransmitters into the brain parenchyma would further increase intracranial pressure (ICP) and therefore form a vicious cycle, resulting in nerve cell dysfunction, and even death with a variety of neurological symptoms, clinical manifestations^11^. Therefore, the destruction of BBB may be the initiation and trigger of SAE. It is been widely accepted that the key to prevent and treat SAE is to maintain the integrity of BBB.

Intraperitoneal hypertension (IAH) and abdominal compartment syndrome (ACS) are the pathological conditions of two different stages of development after various causes of intraperitoneal pressure. Incidence and death rate is extremely high in critically ill patients^12,13^. IAH is one of the common symptoms in sepsis patients. Studies have shown that the incidence of secondary IAH is 39% in sepsis and related septic shock patients^14^. In sepsis, the incidence of IAH is higher, and even some studies believe that IAH with intra-abdominal infection is two of the same kind^15,16^. Interestingly, a study reported recently found that biliary or gastrointestinal origin of abdominal infection occurred significantly increased the risk of SAE^5^. However, the relationship between IAH and SAE is not yet clear so far.

Although it is well known that coughing, defecation and vomiting, which cause elevated abdominal and thoracic pressure, can cause a temporary increase in ICP^17^. Studies have shown that IAH combined with sepsis is more likely to cause increased ICP and significantly reduce the oxygen content of brain tissue^18^. According to the Monroe-Kellie hypothesis^19^, the cranial cavity is non-inflatable, which is filled with brain tissue, cerebrospinal fluid and blood, if the cranial cavity of a material volume increases, the other two substances must adjust their volume to compensate and maintain balance. Cerebrospinal fluid and cerebral venous blood through the jugular vein and excretion of the brain. Elevated IAH may be transmitted to the chest, increased intrathoracic pressure and then push the high jugular vein pressure, thereby affecting the cerebrospinal fluid and blood drainage, the ICP increased and decreased brain perfusion pressure^20–22^. Jarosz B *et al*. in the IAH model found that maintaining the abdominal pressure at 25 mmHg level 90 min in rats, will make the jugular vein pressure increased significantly. Brain tissue magnesium content decreased, while the calcium and zinc ions significantly increased, and observed neuronal atrophy and vacuolization^23,24^. The Study suggested that BBB permeability disorders after IAH caused by electrolyte imbalance, neurons apoptosis and death. Asser M. Youssef’s *et al*.^25^ found that 20 mmHg of IAH for 4 hours will cause temporary, reversible BBB permeability increased, while after 1 hours of abdominal decompression, the function of BBB returned to normal. The results suggested that IAH damage to the central nervous system is closely related to the level and duration of IAH; So, whether there is an internal link between IAH and SAE is an unanswered question.In this study, we hypothesizes that establishment of intraperitoneal hypertension combined with intraperitoneal injection of lps can affect the neuronal structure and function, thereby impact on the central nervous system and trigger SAE.

## Materials and Methods

### Animals

The study was approved by the Animal Protection and Utilization Committee of Xiamen University Xiamen University. All experiments in this study were performed in accordance with relevant guidelines and regulations. A total of 48 BALB/c mice were obtained from department of Veterinary Medicine, Xiamen University. They were randomly divided into four independent experimental groups, as described below. Then, in all four groups after the electrodes were implanted, the neurological function of all mice was evaluated by electroencephalography (EEG).

- Experimental group 1: Twelve mice were divided into intraperitoneal injection of physiological saline group called Control group (n = 12).
- Experimental group 2: Twelve mice were divided into intraperitoneal injection of lipopolysaccharide group called LPS group (n = 12).
- Experimental group 3: Twelve mice were divided into intraperitoneal hypertension group called IAH group (n = 12).
- Experimental group 4: Twelve mice were divided into injection of lipopolysaccharide with intraperitoneal hypertension group, shortened from LPS + IAH group. (n = 12).

### Preparation of Animal EEG Model

Electrodes for recording electroencephalography were placed in each animal by stereotactic surgery 24 hours before induction of sepsis. Intraperitoneal anesthesia with 10% chloral hydrate under the same sedation using the sedation score, then their heads were fixed in a stereotaxic instrument (Stoelting Brain Stereo, USA). After routine sterilization, an arc-shaped incision (3 cm) was made in the top of the head to expose the bregma and other cranial sutures. Topical anesthesia (10% lidocaine) was sprayed onto the skull surface to remove the periosteum. Then drill three holes in the skull and use 0.6 mm sterile stained steel.

### IAH Model and LPS + IAH Model

The LPS model was made by intraperitoneal injection of lipopolysaccharide, and IAH model was the use of intraperitoneal injection of nitrogen. We used an simple and improved pressure gauge detection of abdominal pressure, which to detect and maintain the abdominal pressure to 20 mmHg, for 30 min. In the IAH model, mouse need to be ensure that the heartbeat continued beating, if it death, the experiment would be interrupted and repeat it again. LPS + IAH model was given immediately after intraperitoneal injection of lipopolysaccharide to maintain IAH state for 30 min.

### Establish the occurrence of SAE

**Figure 1:**
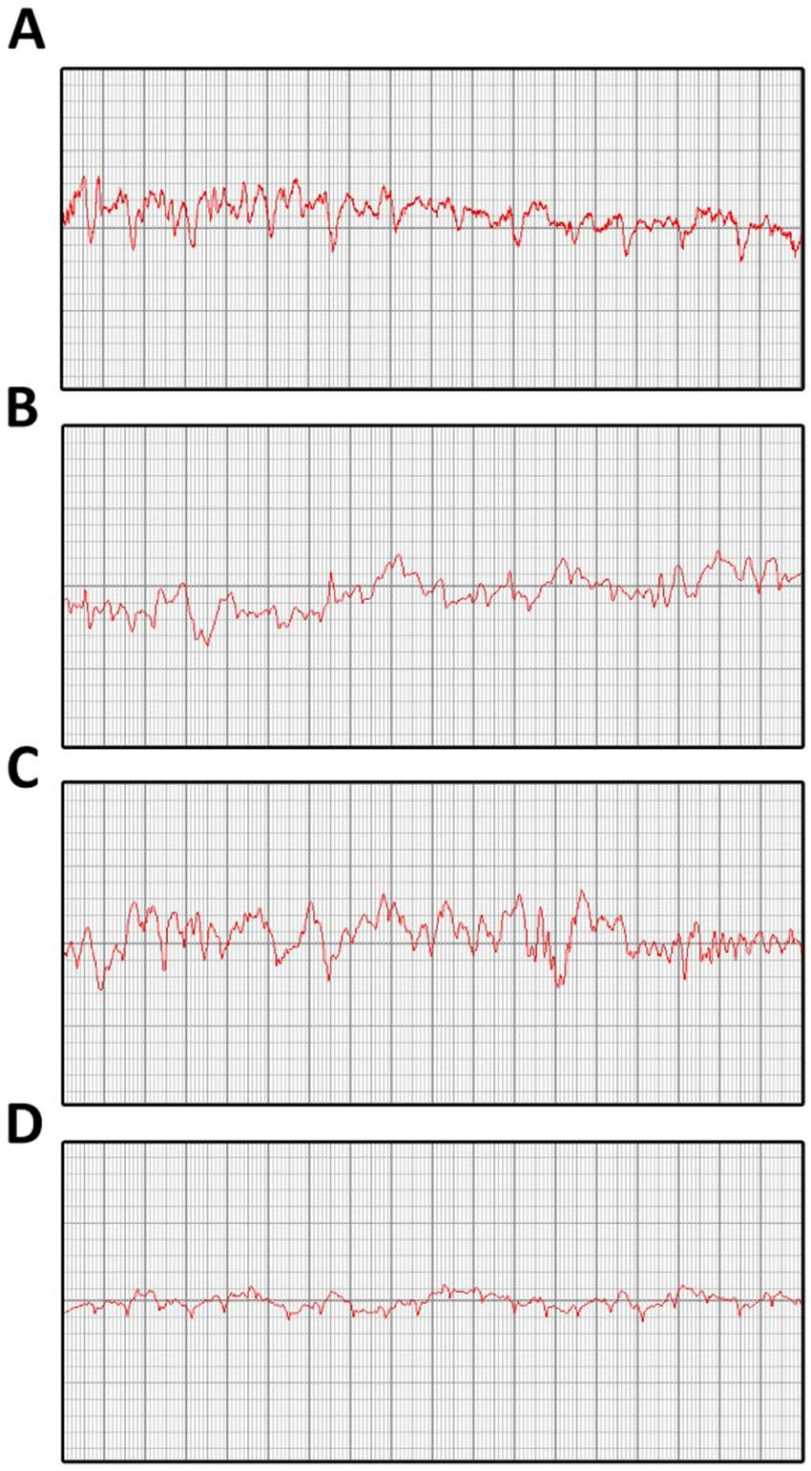
Electroencephalogram result of each group in the study. The δ wave in electroencephalogram of IAH + LPS group after modeling 24 h (**D**) was significantly more frequent than IAH group (**A**), LPS group (**B**) and control group (**C**).

Following these experiments, we established diagnosing SAE by examining EEG tracings recorded using a RM6240 biological signal recorder. Briefly, when delta waves were obvious in the EEG, accompanied by reduced neurobehavior, indicating that SAE occurred in mice, the EEG of each group after the experiments are shown in Fig. 1.

### Brain Tissue Fixation and Sectioning for Light Microscopy

After removal, the brain was dissected according to the brain atlas^26^ resulting in a 0.5 cm × 0.5 cm × 0.1 cm block containing the frontal cortex. The tissue was then immersed in 4% paraformaldehyde immobilized in 0.1 M PBS (pH 7.0–7.6) containing 0.1% sodium diethyl carbonate for 30 minutes and then dehydrated for 30 minutes each in increasing concentrations of ethanol. Tissues were then washed in xylene for 2 × 30 minutes and then embedded in paraffin wax at 55 °C using a copper mold. The brain was then cut at a thickness of 4 μm using a Leica ultrathin microtome (Leica CM1950, Germany) and mounted on polylysine-coated glass slides. The sections were then incubated at 60 °C overnight and then dewaxed by immersion in xylene for 2 × 5 minutes followed by continuous hydrolysis for 2 × 5 minutes in 100%, 95%, 90%, 85% and 75% ethanol. The sections were then washed in tap water and then washed with PBS for 2 × 5 minutes. Then, these sections were stored at room temperature until assayed.

### Western blotting Detection of Bcl-2, Mfsd2a, Cleaved-parp

The brain tissue of the mouse cortex was first removed and the tissue was cleaved with the lysate containing the protease inhibitor. Briefly, the cortex was homogenized on ice using immunoprecipitation buffer (10 mM Tris-HCl, pH = 7.4, 150 mM NaCl, 2 mM EDTA, and 0.5% Nonidet P-40) plus protease inhibitors (1 μg/ml aprotinin, 1 μg/ml leupeptin, and 1 μg/ml pepstatin A). The lysates were collected, centrifuged at 10,000 g for 10 min at 4 °C. The supernatant was removed, and protein concentration was determined using UV spectrophotometer(Beckman Coulter, DU800,American). Equal amounts of protein were separated on 4–12% NuPAGE Novex Bis-Tris gradient gels and transferred to the nitrocellulose membranes. After blocking with 5% non-fat milk for 1 h at room temperature, membranes were incubated with anti- Bcl-2 antibody(1:500; Abcam-Biotech, Abcam, Cambridge, Britain), anti-Mfsd2a antibody(1:500; Sigma-Alorich, Sigma, CA, American),anti-Cleaved-parp antibody(1:500; Abcam-Biotech, Abcam, Cambridge, Britain) overnight at 4 °C, followed by incubation with horseradish peroxidase-conjugated secondary antibodies for 1 h at room temperature. The protein bands were detected by enhanced chemiluminescence and the quantitation of bands was performed using the Image J software.

### Immunohistochemical Detection of Bax; Caspase-3

The sections were first incubated in a 5.1% methanol-hydrogen peroxide solution to quench the endogenous peroxidase for 10 minutes at room temperature and then for 3 × 5 minutes in distilled water and 0.1 M PBS. The sections were then blocked with normal goat serum for 20 minutes at room temperature. The sections were then stained in primary antibody (anti-Bax, rabbit anti-mouse monoclonal, Abcam-Biotech, Abeam, Cambridge, Britain) overnight, then washed with 0.1 M PBS for 3 × 5 minutes. Biotinylated secondary IgG antibody (Fuzhou Meixin Bioengineering Co., Ltd., Fujian Province, China) was then used to incubate at 37 °C for 20 minutes followed by 0.1 M PBS for 3 × 5 minutes. The sections were then incubated in horseradish peroxidase-labeled streptavidin solution for 20 minutes at 37 °C and then for 3 × 5 minutes in 0.1 M PBS. Slides were then prepared using the 3,3’-diaminobenzidine avidin biotin kit as described in the specification (Mai Xin Bioengineering, Fuzhou, Fujian Province, China). The sections were counterstained with hematoxylin to visualize the nucleus. The sections were then dehydrated serially, dialyzed against xylene, mounted on Permount (Fisher, St. Louis, MO), and then capped. The corresponding reference section were stained with HE.

After staining, sections were observed under a BX51 optical microscope (Olympus, Tokyo, Japan). Cells with visible tan particles in the cytoplasm were considered immunopositive for Bax and Caspase-3. Scores of Bax and Caspase-3 expression were double-blindly scored and scored by the product of staining intensity and number of positive cells (15). In short, the lack of a positive cell score was “a”: the score was a deficiency of positive cells, 0,1–10% positive cells scored 1,51–80% scored 3,81–100% scored 4. The staining intensity score was “b”: negative = 0 (−), weak positive = 1 (+), moderate positive = 2 (++), strong positive = 3 (+++). The average of “a × b” scores was used as the final score.

### Statistical Analyses

Continuous variables were compared by a one-way analysis of variance. When there was a significant difference between groups, multiple comparisons were performed using the Student-Newman-Keuls test. Data are presented as means ± SD. All statistical evaluations were bi-directional and p-values less than 0.05 was considered as a significant difference. Statistical analyses were performed using SPSS 22.0 statistical software (SPSS, Chicago, IL).

### Disclosure statement

The authors declare that they have no financial or personal relationships with other people or organizations that could inappropriately influence this work. The authors also declare non-financial competing interests, including personal or professional relations with organizations and individuals.

### Methods statement

The following various study methods are in accordance with the rigorous experimental processes, and in strict compliance with instructions to complete.

## Results

### Significantly change in electroencephalogram result of IAH + LPS group

Compared with the control group, the δ wave in electroencephalogram of the IAH and LPS group are close to the control operation group (Fig. 1A–C) (all P > 0.05). However, IAH + LPS group after modeling 24 h, the δ wave in electroencephalogram was significantly more than other three group (All P < 0.01) (Fig. 1D).

### Decreased neurobehavioral score in IAH + LPS group

**Figure 2:**
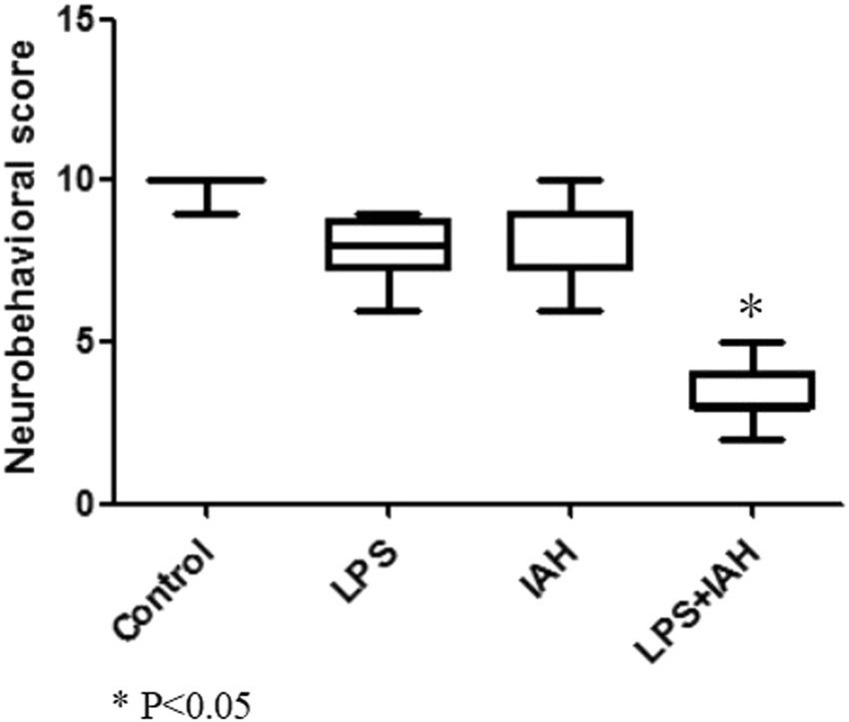
Neurobehavioral score in each group of the study. The neuronal behavioral score in LPS + IAH group was dramatically decreased, significantly lower than control group, LPS group and IAH group. P < 0.05.

According to the diagnosis of SAE, apart from the EGG, we also test the neuronal behavioral score in these different four groups, results show there were no significant differences in the previous three groups, control group, IAH group, LPS group. However, in the LPS + IAH group, the neuronal behavioral score was dramatically decreased as shown in Fig. 2, which prove that mice in LPS + IAH group successfully induce SAE.

### Histopathology change in brain tissue of LPS + IAH group

**Figure 3:**
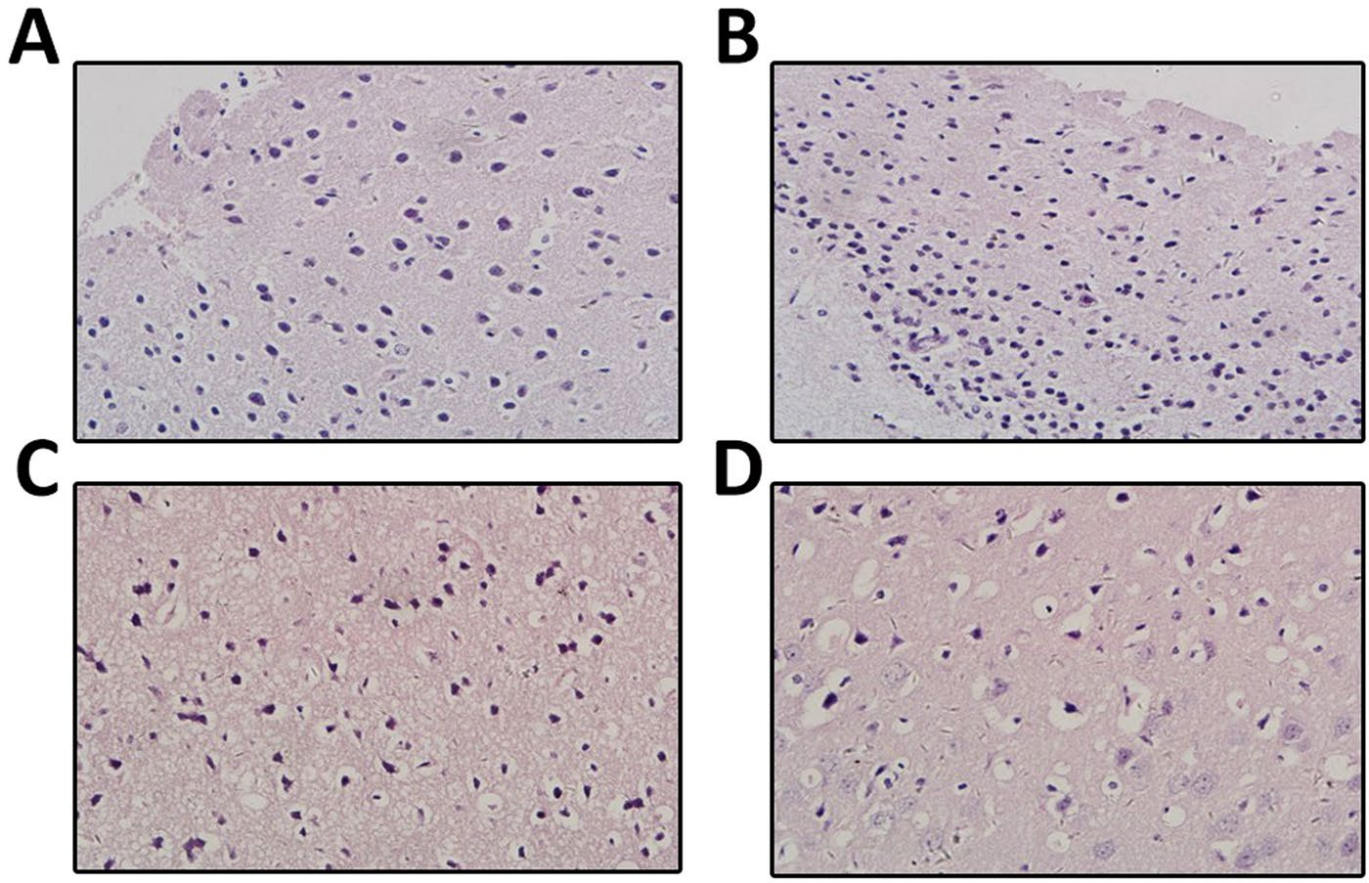
Histopathology change in each group of the study. Normal neural cells with intact cell structure and nuclear membrane integrity was observed in control group (**A**). Neuronal edema increased while the cell structures and nuclear membrane are still intact were observed in LPS group (**B**) and IAH (**C**). Neuronal pyknosis, disintegrated cell structure, plasma membrane breakage, and loss of intracellular contents were observed in LPS + IAH group (**D**).

The control group had shown normal mice neural cells with intact cell structure and nuclear membrane integrity (Fig. 3A). IAH group and LPS group results showed some but no major changes in neural cells morphology and cell structures (Fig. 3B,C). LPS + IAH group results had shown obviously neuronal pyknosis, disintegrated cell structure, plasma membrane breakage, and loss of intracellular contents (Fig. 3D). As compared with control group,the IAH group and LPS group shown that neuronal edema dramatically increased and the cell structures and nuclear membrane are intact. The LPS + IAH group had shown worse damages than any other three group (Fig. 3D).

### Apoptosis observed in LPS + IAH group

**Figure 4:**
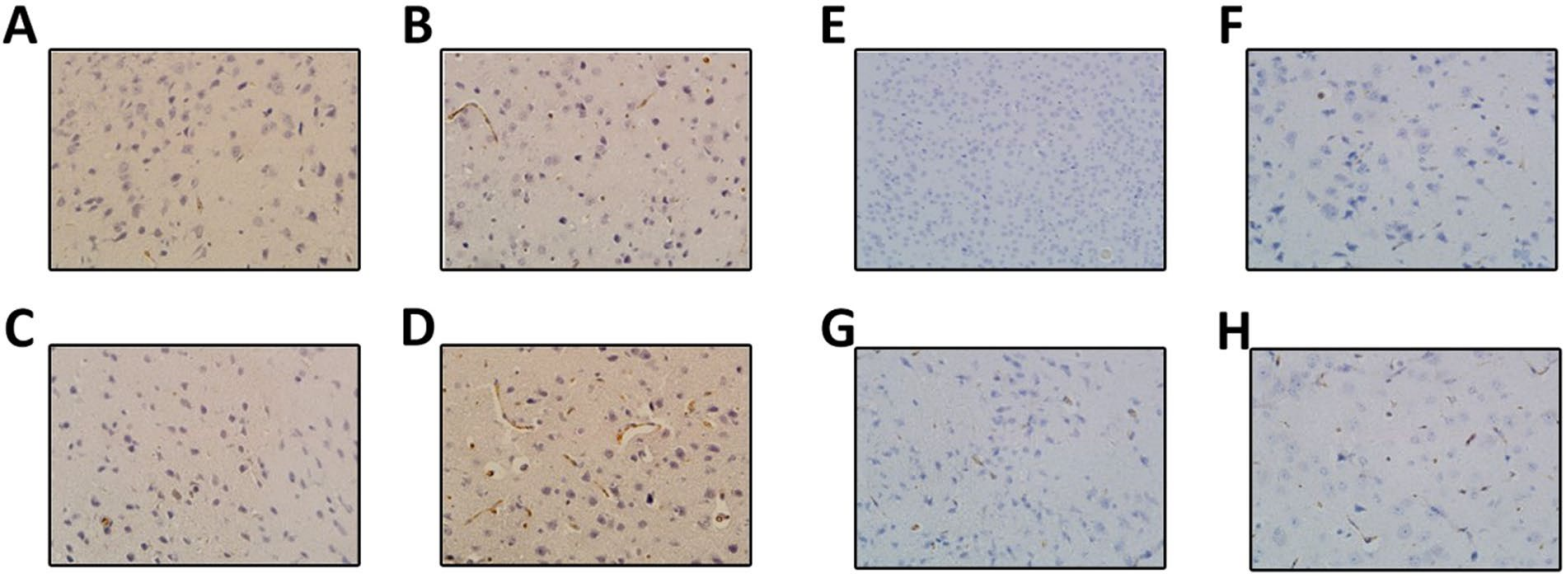
Apoptosis observed in each group of the study. The highest expression level of Caspase-3 was found in the prefrontal cortex of the IAH + LPS group (8.7 ± 0.6) (**D**) than in control group (0.3 ± 0.6) (**A**), LPS group (2.3 ± 0.6) (**B**) and IAH group (0.7 ± 0.6) (**C**). Similarly, the highest expression level of Bax was observed in IAH + LPS group (8.3 ± 0.6) (**H**), and modest expression observed in LPS group (2.7 ± 0.6) (**F**) and IAH group (2.8 ± 0.5) (**G**) and lowest expression level in control group (0.4 ± 0.5) (**E**).

In order to evaluate apoptosis in relative four group, an immunohistochemistry had been performed to assess the expression level of Bax and Caspase-3. The highest Bax(8.3 ± 0.6) and Caspase-3(8.7 ± 0.6) were found in the prefrontal cortex of the IAH + LPS group (Fig. 4). However, there was no significant difference in the level of Bax and Caspase-3 between the control group, IAH group and LPS group (P > 0.05).

### BBB function of LPS + IAH group and more apoptosis observed in LPS + IAH group

**Figure 5:**
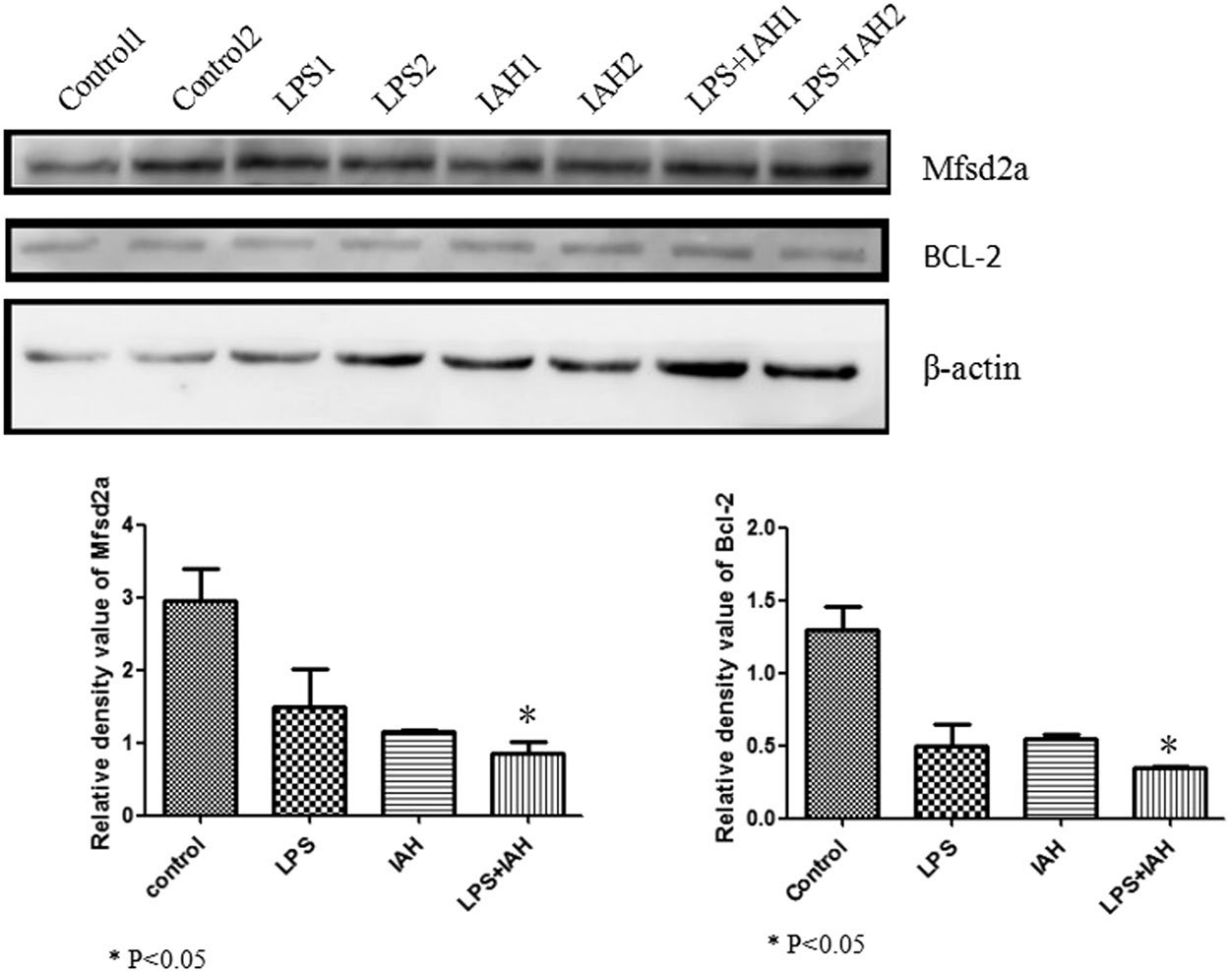
Expression of Mfsd2a and Bcl-2 in each groups. The expression level of Mfsd2a were lower in LPS group and IAH group than control group, while lowest expression in LPS + IAH group (P < 0.05). The expression of apoptosis-related proteins Bcl-2 was found lowest level in the IAH + LPS group.

**Figure 6:**
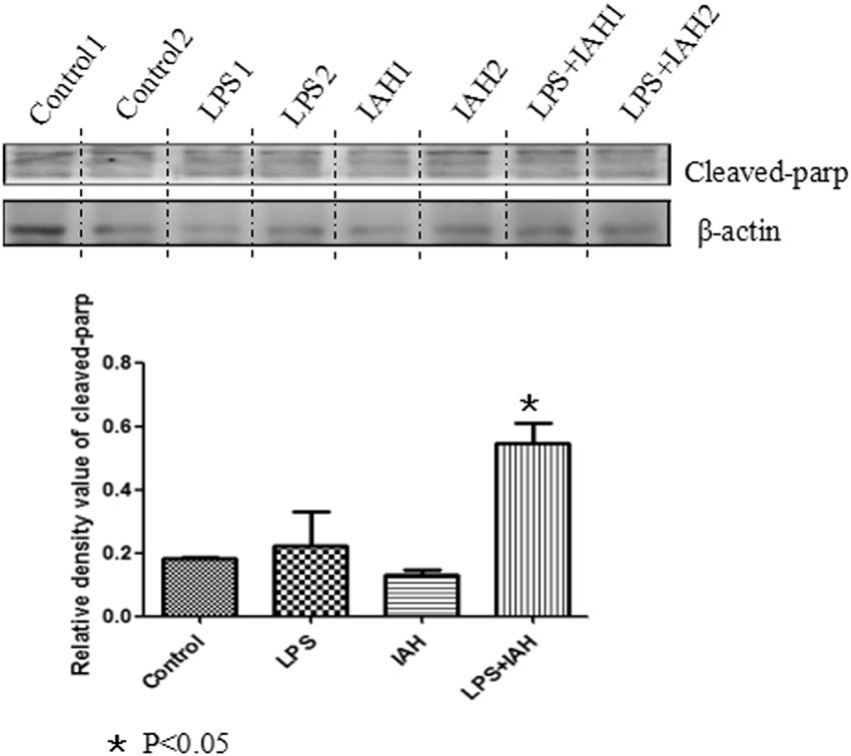
Expression of Cleaved-parp in each groups. The expression of Cleaved-parp was assessed and the expression level in IAH + LPS was the highest (P < 0.05).

To assess the BBB function of mice in experimental group, we evaluate the expression level of Mfsd2a in western blotting. Also shown in Fig. 5, among those four groups, lower expression of Mfsd2a observed in LPS group and IAH group than control group, while lowest expression in LPS + IAH group (P < 0.05). We also detect the expression of apoptosis-related proteins Bcl-2, and found that in the IAH + LPS group, the secretion of Bcl-2 was the lowest. The expression of Cleaved-parp was also assessed and the expression level in IAH + LPS was the highest (Fig. 6) (all P < 0.05), which further showed that apoptosis did occur in IAH + LPS group.

## Disscussion

The mechanistic explanation for the IAH negative cerebral impact remains controversial. Many reasons including physical factor, gut bacterial translocation and systemic inflammatory response may contribute to the impact. In general^9^, the vascular endothelial cells that make up BBB have a special transport system responsible for the selective transport of various substances required by the central nervous system through the BBB. It is generally accepted^27^ that the barrier function of BBB is mainly performed by the tight junctions between cerebrovascular endothelial cells and their cells. There is almost no gap between the cerebrovascular endothelial cells, which are connected by the junctional complex consisting of the tight junction at the top of the cell and the adhesion junction at the base of the cell, respectively. Tight junction is a protein complex composed of multiple proteins, including transmembrane proteins, cytoplasmic adhesion proteins, adhesion molecules and cytoskeletal proteins. Among these transmembrane proteins are Occludin and the Claudins family on endothelial cells; these transmembrane proteins are linked to the cytoskeleton via cytosolic proteins ZO-1/ZO-2/ZO-3^28^. Under normal circumstances, tightly connected structure maintains a dynamic balance of adjustment, its main functions include: (1) to close the gap between cells, play its permeability regulating function, limiting the movement of ions and proteins through the organization. (2) Tight junctions are involved in the maintenance of cell polarity, forming chemical and ionic gradients between the cell monolayers, thereby facilitating vector transport. The changes of BBB function are related to the opening and closing of tight junctions, and the tight junctions and opening are closely related to the expression of related proteins. Various types of exogenous stimulation of vascular endothelial cells tightly linked to specific proteins and cytoskeletal protein structure dysfunction, changes in location, protein expression decreased the destruction of tight junction protein integrity, can cause its dysfunction, causing BBB permeability sexual increase^29^. Therefore, the integrity of tight junctions between vascular endothelial cells plays an important role in maintaining endothelial polarity and regulating endothelial cell permeability. We hypothesize that in sepsis, in addition to physical factors, changes in intraperitoneal pressure may cause abnormalities in tight junction-related protein expression that may affect BBB integrity.

In some cases, although some rat BBB vascular endothelial cell tight junctions are intact, they also show obvious edema of glial foot process. why? Are there other factors that increase the permeability of vascular endothelial cells beyond tight junctions? Perhaps the major facilitator superfamily of domains recently discovered by Gu’s team, MFSD2a, seems to explain this phenomenon^27^. They are concerned with the second barrier crossing mechanism: transcytosis, in which some substances are transported through the barrier cells by vesicles. Cytotriasis often occurs in other parts of the body, usually in the BBB was inhibited. Mfsd2a may be one of the inhibitors. They studied the development of BBB and found that the critical time window for BBB formation in mice was 15.5 days at embryonic day. Screening of BBB-specific gene expression at this crucial time revealed that Mfsd2a is specifically expressed in blood vessels formed by the BBB. Knocking out the Mfsd2a gene results in the destruction of the BBB from embryo to all stages of adult life, but normal vascular function is normal. Electron microscopy found that endothelial cells lacking the Mfsd2a gene showed enhanced efflux of vesicular vacuoles, but no obvious defects in tight junctions. These studies found that Mfsd2a is a switch in the permeability of BBB endothelial cells that, in the absence of such molecules, can be transported by rapid swallow transport of endothelial cells in the presence of this molecule, which is significantly suppressed by endothelial cell trafficking. Thus,whether the abnormality of Mfsd2a expression is critical for blood-brain barrier damage, cerebrovascular endothelial cells Mfsd2a abnormal expression occurs in SAE? Abdominal pressure on it impact? We hypothesize that by studying the key BBB-associated brain tissue proteins of Mfsd2a, combined with changes in brain function and cerebral cortical microstructure, could found the key point of SAE occurs when IAH binds LPS.

So what is the mechanism between IAH and BBB damage? We hypothesize that in the process of sepsis, IAH often accompanied by different degrees of IAH, intra-abdominal pressure increases the internal jugular vein pressure, BBB capillary lumen pressure also increases, endothelial cells feel the pressure changes, first of all caused the BBB permeability related protein function or expression and distribution abnormalities such as the key protein, Mfsd2a to make the permeability of BBB increase, a large amount of water and various cytokines, pro-inflammatory cytokines, pseudo-neurotransmitters, etc. enter the brain parenchyma, causing brain damage and trigger SAE. When the abdominal pressure reaches a certain degree and lasted for a certain period of time, the gap junction is damaged due to the serious damage of the structural protein, then a large amount of neurotoxic substances will flow into the brain, resulting in a sharp deterioration of SAE. If IAH is controlled in time, the related protein abnormalities can be reduced or avoided, the damage of BBB permeability can be alleviated, and the occurrence of SAE can be delayed or avoided.

Sepsis-associated encephalopathy is a serious disease, which have poor prognosis. Previous studies suggest that IAH may increase BBB permeability and may be associated with the pathogenesis of sepsis encephalopathy, but the specific mechanism is unclear. Our results demonstrated that IAH can promote the presence of SAE in SE mice, and its possible mechanism is to induce the occurrence of SAE by promoting neuronal apoptosis and impact on the BBB. IAH resulting in BBB damage and increased permeability is also confirmed by other animal experiments^30^, but their study did not specifically detect the relevant BBB protein expression changes.

According previous study reported, many of the causes of sepsis encephalopathy, most of which eventually lead to the occurrence of neuronal apoptosis. And here the neuronal apoptosis we think occurs in the cerebral cortex as the literature points out that major sepsis encephalopathy of symptoms such as neuronal microglia inflammatory response or BBB destruction, presence in cortical neurological deficits. The main symptoms of SAE are delusional, psychiatric changes, severe coma, etc^31^. The hippocampus is mainly responsible for learning, memory, etc, while the cortical neurons are responsible for the emotional, spiritual, etc^32^. As the most common symptom of sepsis encephalopathy is delirium^33^, so we focus on the related protein expression changes on cortical neurons. For instance, sunyanli, *et al*.^34^ found that intestinal microecological conditions’ change can also induce septic encephalopathy, although their main focus was on the vagal function, the cortical neurons glial cell inflammatory response to the pathogenesis of sepsis encephalopathy was studied. Previous studies have shown that electroencephalogram in patients with sepsis encephalopathy has its characteristic changes^35^. In general, we use α, β, θ, δ four waveforms to describe the pathophysiological state of the brain, α, β waves can be found in patients with clear and sleep, while δ wave prone to patients with sepsis encephalopathy^36^. In our experiment,as shown in Fig. 1, four groups of mice were recorded in the EEG, a significant increased delta wave could be found in SAE group,while the other three groups were more α or β wave.Neuronal behavior score decreased accompanied by δ wave increased in EEG is a necessary condition for the diagnosis of sepsis encephalopathy.

Throughout the experiment, we studied the electroencephalogram, the cellular structure of mouse cortex, and key proteins associated with the blood-brain barrier. First, we studied the electroencephalogram to confirm that we successfully induced SAE under our experimental system conditions. We have increased the credibility of the experiment through neurobehavior and EEG validation. In order to study the key proteins involved in the cerebral cortex and the blood-brain barrier, we believe that they are very closely linked and ultimately result in the destruction of the blood-brain barrier due to changes in the micro-structure of the cerebral cortex. Therefore, we focus on the study of cortical neurons.

We have demonstrated that sepsis encephalopathy does occur in BALB/c mice after successful induction of SE by lps with the retention of IAH for 30 min after 24 h later, which indicated that LPS accompanied by IAH promote the occurrence of sepsis encephalopathy.

The most classic for the induction of sepsis is Cecal Ligation and Puncture (CLP)^37^, but for our experimental design, CLP may not be suitable for follow-up IAH experiment. In our experimental system, if the CLP experiment is conducted, it is impossible to distinguish whether the eventual SAE occurs will be caused by the CLP itself or by the experimental system that we envisaged to establish. Therefore, we believe that the establishment of sepsis by intraperitoneal injection of LPS syndrome model, combined with IAH, is a way of improving and not being criticized. We summarize the previous animal experimental conditions, by intraperitoneal injection of lps to induce SE. Through our observation, the body temperature decreased, activities decreased, eye pus discharge increased generally occurs in intraperitoneal injection lipopolysaccharide 2–3 hours later, the most common is the emergence of diarrhea.

After establishing lps-induced sepsis and intraperitoneal hypertension combined with lps-induced sepsis encephalopathy, we intend to further find its possible mechanism. We first conducted tissue HE detection and found that LPS + IAH group of cerebral cortical neurons in the structural changes than the other three groups were more obvious, mainly for neuronal disorders, neuronal cell morphology, and even neuronal cell shrinkage/disruption/fragmentation. It was proved that in the LPS + IAH group, neurons are likely to undergo apoptosis. Therefore, we further detect the apoptosis-related classical protein by western-blotting and immunohistochemistry. It can be found that the expression of Bcl-2 by western blotting in LPS + IAH group was significantly decreased, and the content of Cleaved-parp protein in LPS + IAH group was significantly higher than the other three groups (P < 0.05). And the expression of Bax in LPS + IAH group was significantly increased by immunohistochemistry, while the increased of immunohistochemical grade of Caspase-3 was observed, All above indicated that neuronal apoptosis occurred in LPS + IAH group. Interestingly, the secretion level of Mfsd2a, the blood-brain barrier related important protein, was also detected. Blood-brain barrier disruption should not be limited to Mfsd2a expression. However, Mfsd2a is an important key protein in addition to tight junctions that are highly correlated with vascular endothelial cell permeability, since in our experiments we explained that the destruction of BBB induced by IAH is caused by increased pressure in the jugular vein, lead to BBB capillary pressure increases, accompanied by a large number of inflammatory cytokines into the brain parenchyma, causing BBB damage. Sustained inflammation always leads to organ damage and function impaired^38–42^. In addition, in published paper^25^, the authors used intraperitoneal injections of mineral oil to establish an intra-abdominal pressure model, followed by iv injection of iridol-blue and macroscopic observation of brain parenchyma. After a period of time, iridol-blue was observed to enter the parenchyma, indicating that the BBB was damaged after establishing the model of abdominal pressure. The article further confirmed that IAH can cause BBB damage.

In our observation,the secretion of Mfsd2a in IAH group was significantly lower than that in control group (P < 0.05) compared to the other three groups.That is to say, intraperitoneal hypertension itself may cause damage to the blood-brain barrier, but with the clear detection of apoptosis-related protein levels, the neuronal apoptosis event occurred only in the LPS + IAH group. Although Bcl-2 in the IAH group also appeared to reduce significantly, which itself reduction can not fully represent whether the occurrence of apoptosis. But in the Bax, especially the key apoptotic proteins, such as Caspase-3 and Cleaved-parp analysed in our study, IAH group had no significant difference compared with the control group, which was also consistent with animal neurobehavioral changes in EEG.

Another key point is that we choose nitrogen as the material for establishing the intra-abdominal pressure model because it is an inorganic substance and nitrogen does not act on the abdominal cavity to produce high intra-abdominal pressure. This is a purely physical effect. We originally intended to use air jets, but due to confounding factors, the composition of the air is more complex, such as oxygen, carbon dioxide, we can not tell the end result of the experimental system is due to the air caused by excessive composition, after careful consideration, we choose nitrogen peritoneal cavity injection.Our study still have many deficiencies, although the structure of cortical neurons, and apoptosis-related protein expression in IAH + LPS group obvious change, but whether it also impact on the protein coding process, or IAH + LPS group alter the intracranial pressure, or it also lead to BBB damage on other key protein. we did not draw the appropriate conclusions; But still through our experiments, we are convinced that the physical factors in the abdominal cavity promoting the occurrence of sepsis encephalopathy in mice, which provide inspiration for our future clinical work. In the treatment of sepsis patients, we should pay more attention to monitoring and reduce the abdominal pressure, which is conducive to the prevention and treatment of sepsis encephalopathy.

## Conclusion

Intraperitoneal hypertension (IAH) can exacerbate and increase incident rate of sepsis-associated encephalopathy in sepsis mice by promoting neuronal apoptosis and destruction of the blood-brain barrier.

## Electronic supplementary material

Supplementary Information
