# Supplementary material for: Intraperitoneal hypertension, a novel risk factor for sepsis-associated encephalopathy in sepsis mice
Source: Sci Rep. 2018 May 25;8:8173. doi: 10.1038/s41598-018-26500-7 (PMC5970176; doi:10.1038/s41598-018-26500-7)
Supplement: Supplementary file 1 — Supplementary Information [file 41598_2018_26500_MOESM1_ESM.pdf]

Intraperitoneal hypertension, a novel risk factor for sepsis-associated encephalopathy in sepsis mice

Yu-jing He[1*], Hao Xu[1*], Yao-jie Fu[2], Ji-yan Lin[2], Min-wei Zhang[1]

[1]Intensive Care Unit, First Affiliated Hospital of Xiamen University, Xiamen, Fujian province, China.
[2]Emergency Department, First Affiliated Hospital of Xiamen University, Xiamen, Fujian province, China.

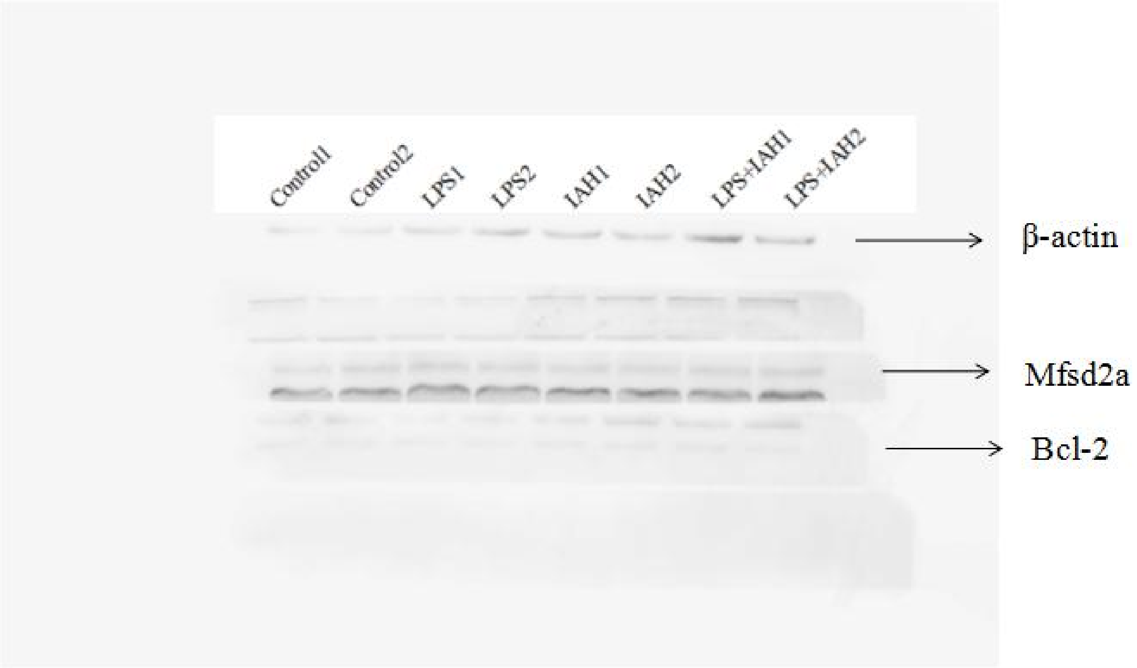
Control1
Control2
LPS1
LPS2
IAH1
IAH2
LPS+IAH1
LPS+IAH2
β-actin
Mfsd2a
Bcl-2

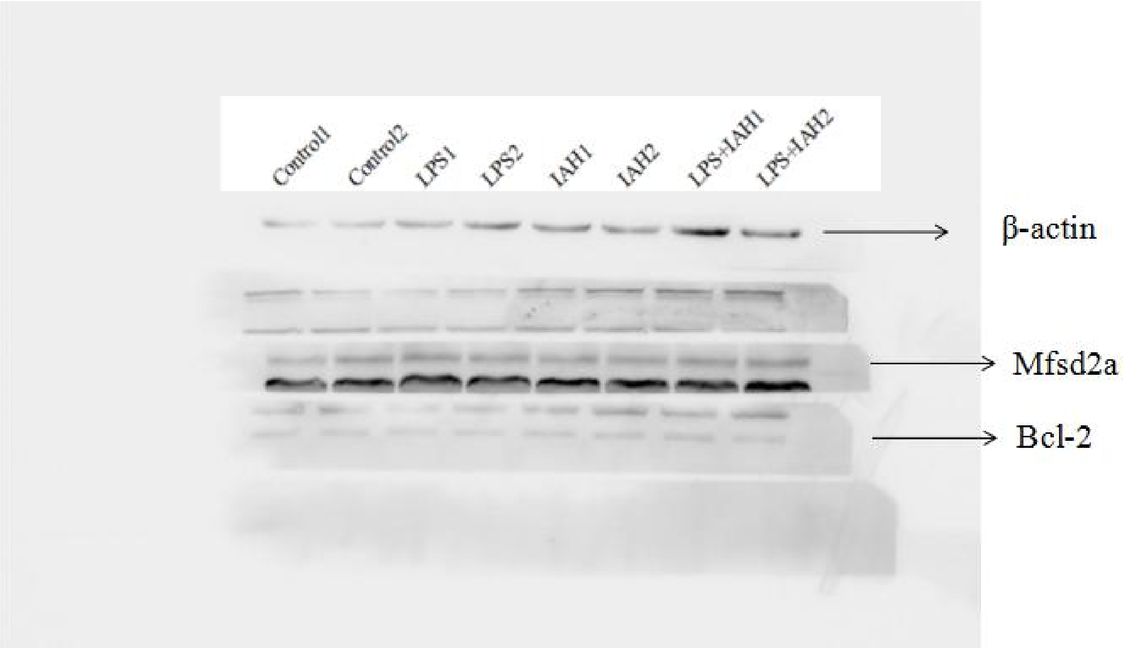
Control1
Control2
LPS1
LPS2
IAH1
IAH2
LPS+IAH1
LPS+IAH2
β-actin
Mfsd2a
Bcl-2

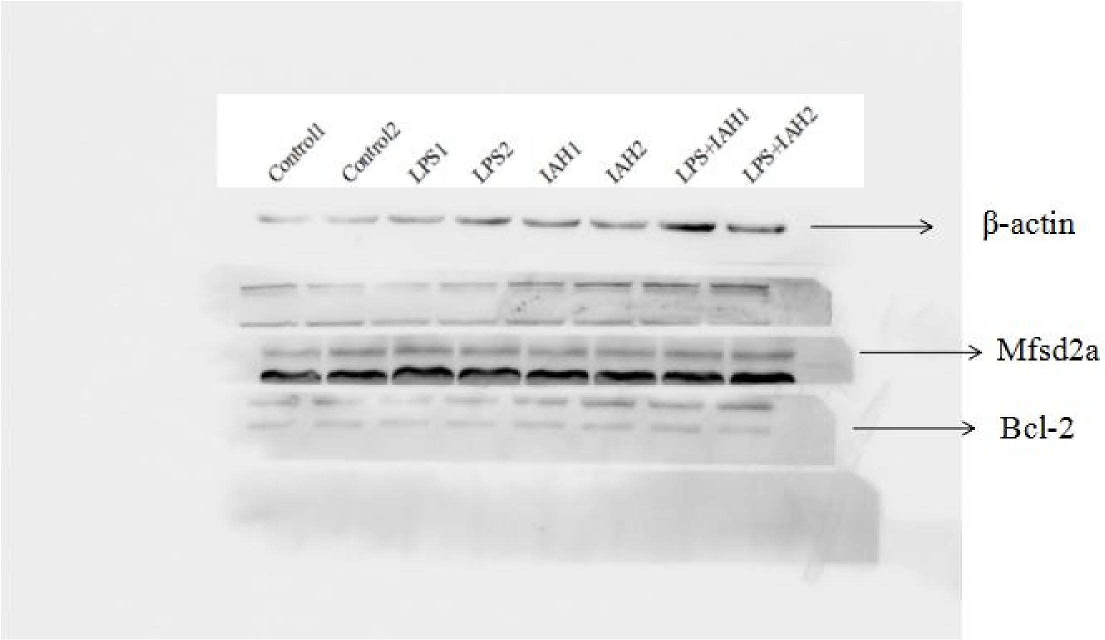
Control1
Control2
LPS1
LPS2
IAH1
IAH2
LPS+IAH1
LPS+IAH2
β-actin
Mfsd2a
Bcl-2

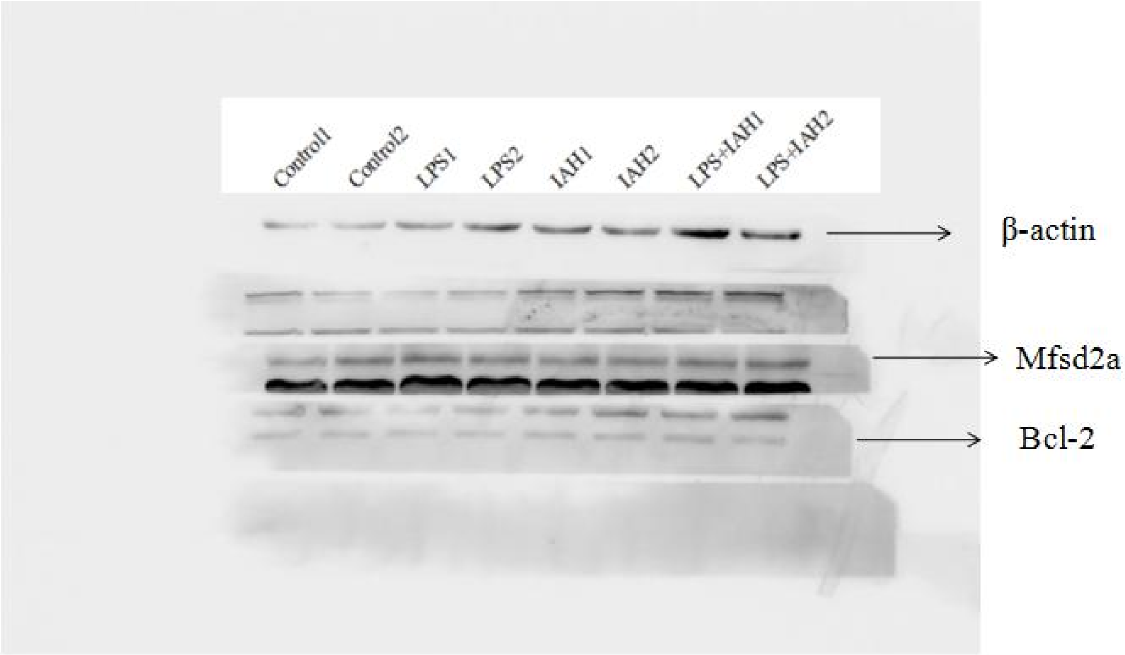
Control1
Control2
LPS1
LPS2
IAH1
IAH2
LPS+IAH1
LPS+IAH2
β-actin
Mfsd2a
Bcl-2

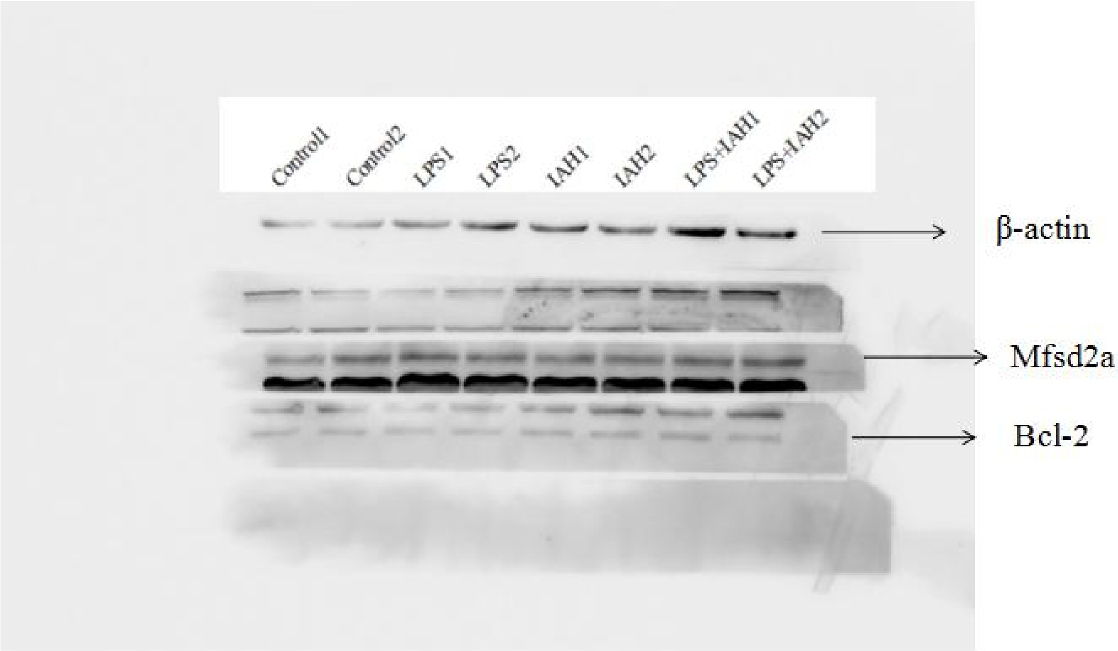
Control1
Control2
LPS1
LPS2
IAH1
IAH2
LPS+IAH1
LPS+IAH2
β-actin
Mfsd2a
Bcl-2

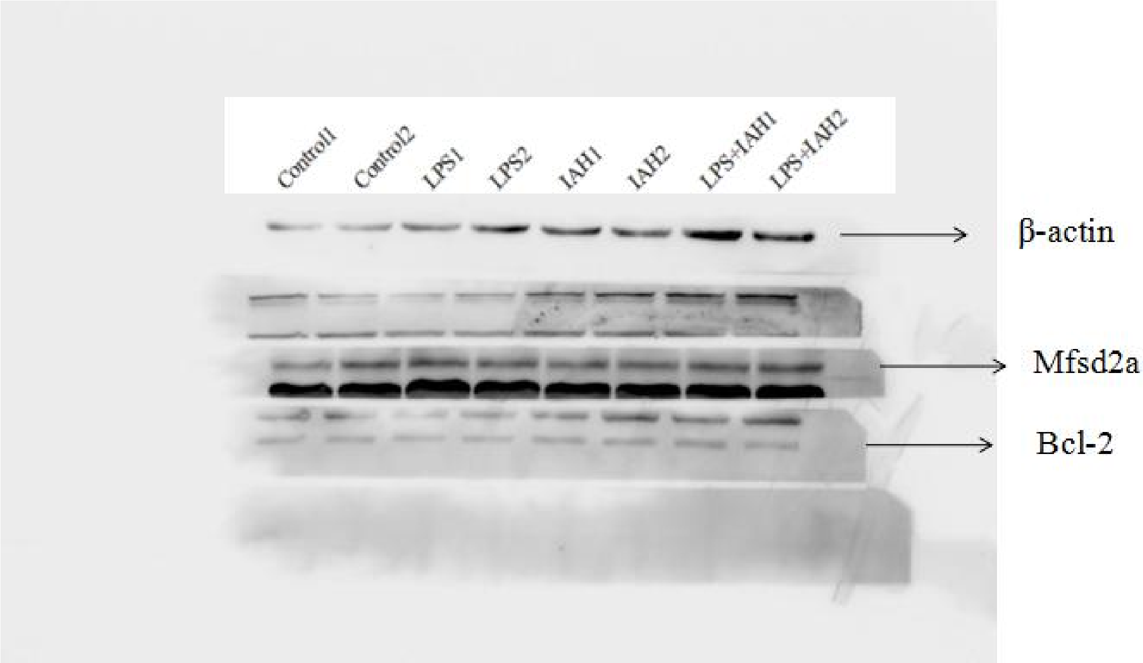
Control1
Control2
LPS1
LPS2
IAH1
IAH2
LPS+IAH1
LPS+IAH2
β-actin
Mfsd2a
Bcl-2

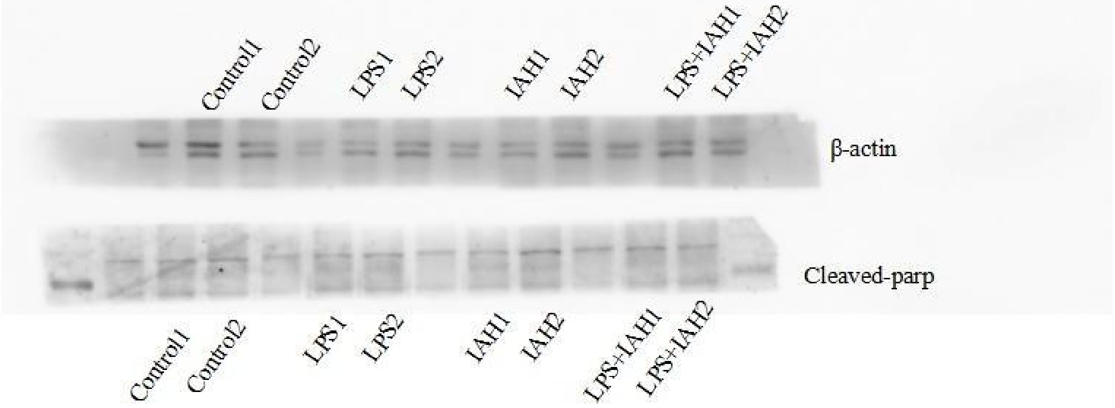
Control1
Control2
LPS1
LPS2
IAH1
IAH2
LPS+IAH1
LPS+IAH2
β-actin
Cleaved-parp
Control1
Control2
LPS1
LPS2
IAH1
IAH2
LPS+IAH1
LPS+IAH2

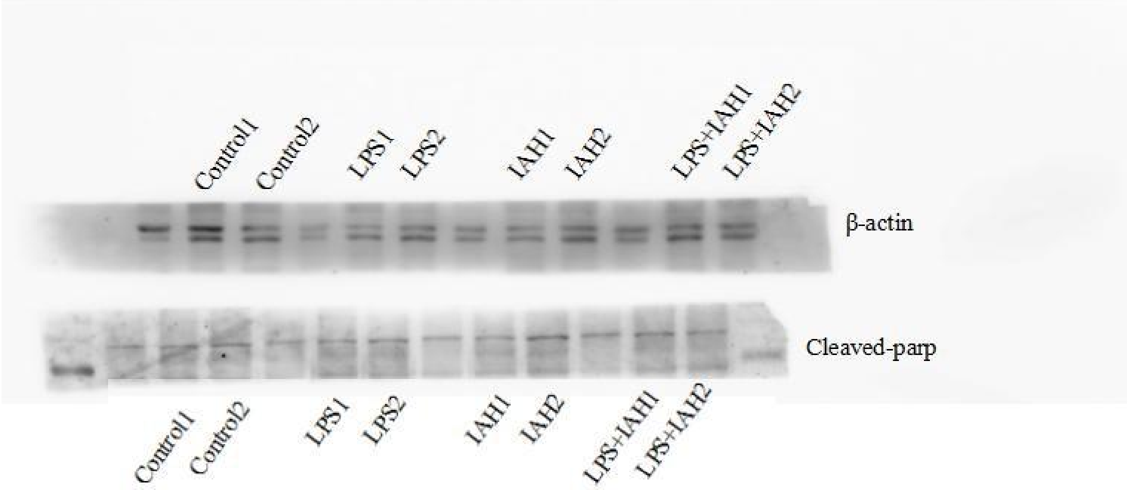
Control1
Control2
LPS1
LPS2
IAH1
IAH2
LPS+IAH1
LPS+IAH2
β-actin
Cleaved-parp
Control1
Control2
LPS1
LPS2
IAH1
IAH2
LPS+IAH1
LPS+IAH2

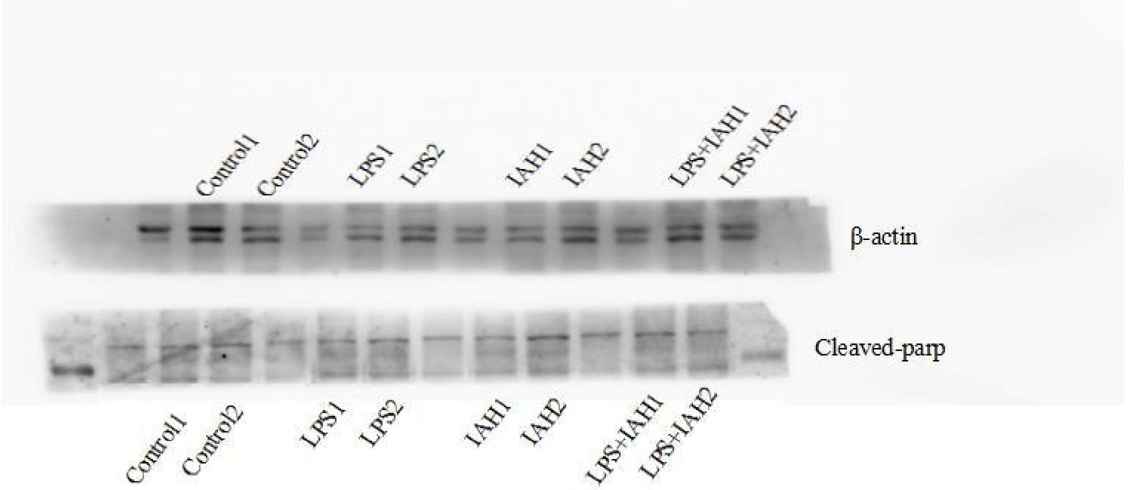
Control1
Control2
LPS1
LPS2
IAH1
IAH2
LPS+IAH1
LPS+IAH2
β-actin
Cleaved-parp
Control1
Control2
LPS1
LPS2
IAH1
IAH2
LPS+IAH1
LPS+IAH2
